# Combination of White Blood Cell Count to Mean Platelet Volume Ratio and Neutrophil-to-Platelet Ratio Predicts Long-Term Adverse Events in Patients with MINOCA

**DOI:** 10.1155/2022/5642406

**Published:** 2022-08-16

**Authors:** Ayman A. Mohammed, Lu Liu, Redhwan M. Mareai, Abdul-Quddus Mohammed, Guoqing Yin, Shekhar Singh, Yawei Xu, Fuad A. Abdu, Wenliang Che

**Affiliations:** ^1^Department of Cardiology, Shanghai Tenth People's Hospital, Tongji University School of Medicine, Shanghai, China; ^2^Department of Cardiology, Shanghai Tenth People's Hospital Chongming Branch, Shanghai, China

## Abstract

**Background:**

White blood cell count to mean platelet volume ratio (WMR) and neutrophil-to-platelet ratio (NPR) have been demonstrated as prognostic inflammatory biomarkers of the acute coronary syndrome. We aimed to evaluate the prognostic value of WMR and NPR among myocardial infarction with nonobstructive coronary arteries (MINOCA) patients.

**Method:**

A total of 274 MINOCA patients were enrolled. Baseline clinical data, blood cell panel, and biochemical parameters were evaluated. The patients were classified according to the medians of WMR and NPR. The primary endpoint of the present study was major adverse cardiovascular events (MACE). Multivariable Cox regression analysis was used to assess the effect of independent variables of WMR and NPR on the dependent variable (MACE).

**Result:**

The median values of WMR and NPR were 701 and 0.03, respectively. During the median follow-up of 28 months, a total of 58 incidences of MACE occurred. The MACE rate was more frequent in high WMR and high NPR patients. In Kaplan-Meier analysis, the incidence of MACE was higher in WMR>701 and NPR>0.03 (long-rank *P* = 0.004 and *P* = 0.002, respectively). The combined high WMR and high NPR showed a significantly higher rate of MACE (long-rank *P* = 0.001). Cox regression analysis showed that the combined high WMR and high NPR were independent predictors of long-term MACE with the highest hazard ratio (HR, 2.511; 95% CI, 1.271 to 4.960; *P* = 0.008).

**Conclusion:**

High WMR and NPR separately or in combination were correlated with increased risk of MACE among MINOCA patients, suggesting WMR and NPR may assist as a reliable inflammatory marker in risk prediction of MINOCA patients.

## 1. Introduction

Globally, ischemic heart disease remains a major cause of death, and its prevalence constantly increases [1]. Around 5–15% of acute myocardial infarction (AMI) patients show nonobstructive (<50% stenosis) coronary arteries, named myocardial infarction with nonobstructive coronary arteries (MINOCA). MINOCA is a diverse entity with an inflammatory etiopathogenesis, in which atherosclerotic plaque disruption is a frequent cause [2–5]. MINOCA remains an interest clinically, which has increased attention over the last decade. Earlier, MINOCA was reported to be a benign entity, but later it was linked with poor clinical outcomes and a high risk of in-hospital mortality [6–9]. Data that might help identify patients at potential risk in acute settings of MINOCA are still limited. Therefore, it could be of potential interest to identify new predictive biomarkers of adverse outcomes that might help the clinician determine whether management strategies translate into amelioration of cardiovascular endpoint in MINOCA patients.

Inflammatory cells (leukocytes) and platelets play a crucial role in the pathogenesis and progression of atherosclerosis and AMI, producing intense inflammatory reactions [10–14]. White blood cell (WBC) count and its subtype have been investigated to predict cardiovascular outcomes in coronary artery disease (CAD) patients as inflammatory markers [15]. The increased neutrophil count is associated with a higher risk of cardiovascular events, and a low lymphocyte count may reflect systemic stress and immunodeficiency, which has been associated with cardiovascular complications and increased mortality rates [16, 17]. In addition, white blood cell count to mean platelet volume ratio (WMR), neutrophil-to-platelet ratio (NPR), platelet-to-lymphocyte ratio (PLR), and neutrophil-to-lymphocyte ratio (NLR) are four easily calculated inflammatory biomarkers from blood cell counts that have been shown to predict unfavorable clinical outcomes in a variety of cardiovascular illnesses, including in AMI [18–23]. Recent clinical studies suggest that MINOCA individuals may also have a proinflammatory predisposition, as seen by several inflammatory biomarkers [24] or more common inflammatory conditions [25]. However, to date, the information on the association between the inflammatory biomarkers and clinical outcomes among the MINOCA population is still limited.

Therefore, the present study aimed to explore the association and prognostic values of inflammatory biomarkers (WMR and NPR) for the first time in acute settings of patients with MINOCA.

## 2. Methods

### 2.1. Study Population

This was a single-centered retrospective observational study that enrolled patients with AMI who underwent invasive coronary angiography (CAG), with new-onset chest pain with an ECG presentation of STEMI and NSTEMI at the cardiology department of Shanghai Tenth People's Hospital (Tongji University, Shanghai, China), during the period from 2014 to 2019.

MINOCA was diagnosed if a patient fulfilled the AMI criteria in the absence of obstructive coronary arteries (type I: <50% stenosis; or type II: no stenosis) as recommended by the recent American Heart Association statement that introduces an updated definition incorporating the 4th universal MI definition, which by agreement excludes Takotsubo syndrome and myocarditis from the eventual diagnosis of MINOCA [2]. Patients who had a previous history of MI or coronary revascularization (PCI or bypass surgery) with acute/chronic infection, autoimmune diseases, systemic inflammatory disease, on treatment therapy which critically affects inflammatory cell count, patients with malignancy, chronic kidney disease, severe liver disease, no available complete blood cell counts, severe anemia, and fever (>38°C) on admission were excluded.

The present study was in accordance with the Declaration of Helsinki and was approved by the hospital's ethical review board (Shanghai Tenth People's Hospital, Tongji University, Shanghai, China), and the informed consent was signed by each participant.

### 2.2. Data Collection

The baseline and demographic clinical data [age, gender, smoking, and comorbidities (hypertension, diabetes, hyperlipidemia, and atrial fibrillation)] were collected from hospital records during admission. In addition, all cases underwent the invasive CAG, an echocardiographic assessment, and a serial electrocardiogram on hospital entry. Comorbidities such as diabetes mellitus, dyslipidemia, and hypertension were diagnosed as previously known, receiving specific therapy or fulfilling the well-established diagnostic protocols on workup during admission. All patients were initially managed with secondary preventative treatments recommended by the latest guidelines.

### 2.3. Biochemical Assessment

Total WBC, neutrophils, lymphocytes, platelets, mean platelet volume (MPV), and other hematologic parameters were measured by an automated complete blood cell count. The present study used the full blood cell panel with the highest WBC count during the first 48 hours of admission to calculate the WMR (as the ratio of WBC count to MPV), NPR (as the ratio of neutrophils to platelets count), PLR (as the ratio of platelets to lymphocytes count), and NLR (as the ratio of neutrophils to lymphocytes count) from the same blood sample test report. The other laboratory parameters were measured and decided according to standard protocols.

### 2.4. Follow-Up and Endpoints

The median follow-up duration for this study was 28 months. Follow-up information was achieved through telephone calls to the patients or their relatives, checking computerized medical records and clinical notes obtained by expert cardiologists to assess the patients' medical status and outcome events or the 1st documented outcome case at the hospital. The primary endpoint of the present study was major adverse cardiovascular events (MACE), including (1) cardiovascular death (defined as the death attributed by cardiac cause, i.e., ACS, fatal arrhythmias, refractory heart failure, or sudden death with no apparent cause); (2) heart failure [26]; (3) nonfatal MI (defined as “rise and/or fall” cardiac troponin biomarkers with symptoms of cardiomyocyte ischemia or the existence of ischemic new ECG changes [27]); (4) angina rehospitalization (defined as any readmission to hospital or emergency department caused by anginal chest discomfort with recorded evidence by a physician); and (5) stroke (defined as evidence of cerebral ischemic infarct caused by thromboembolic occlusion or intracranial hemorrhage) [28].

### 2.5. Statistical Analysis

All analyses in this study used SPSS (v.22), and the figures were framed by GraphPad software (version 8.0.1; Inc., USA). The mean ± standard deviation (SD) was used for numerical variables, and percentages were used for categorical data. Comparisons between groups of continuous variables were assessed by analysis of variance (ANOVA), while the categorical variables were expressed as the number of participants with percentage (%) and compared by the chi-square (*x*^2^) and Fisher's exact tests. The study population data analysis was classified according to the combination of WMR and NPR median values. The correlation between WMR and NPR was analyzed using the Spearman correlation coefficient. To determine the effect of independent variables of WMR and NPR on the dependent variable (MACE), Cox proportional regression models were constructed to obtain hazard ratio (HR). Multivariable Cox regression models were adjusted for traditional cardiovascular causative factors that are known to be associated with increased risk of MACE for MINOCA patients, such as age, sex, smoking, hypertension, diabetes mellitus, hyperlipidemia, atrial fibrillation, left ventricular systolic ejection fraction (LVEF), and the degree of coronary stenosis. To estimate the MACE-free survival, a Kaplan-Meier analysis was performed, and the log-rank test was used to determine the dissimilarity between groups. The receiver operating characteristic (ROC) graph was used to evaluate the significance of NPR and WMR in predicting the clinical outcomes. We also performed interaction term analysis to investigate the independent risk associated with WMR and NPR in specific patient subgroups [age (<65 years or ≥65 years), sex (male, female), and comorbidities (hypertension, diabetes mellitus, smoking, and atrial fibrillation)]. Statistical analyses performed were 2-sided; a *P* < 0.05 is considered significant.

## 3. Results

### 3.1. Clinical Baseline Characteristics

**Figure 1:**
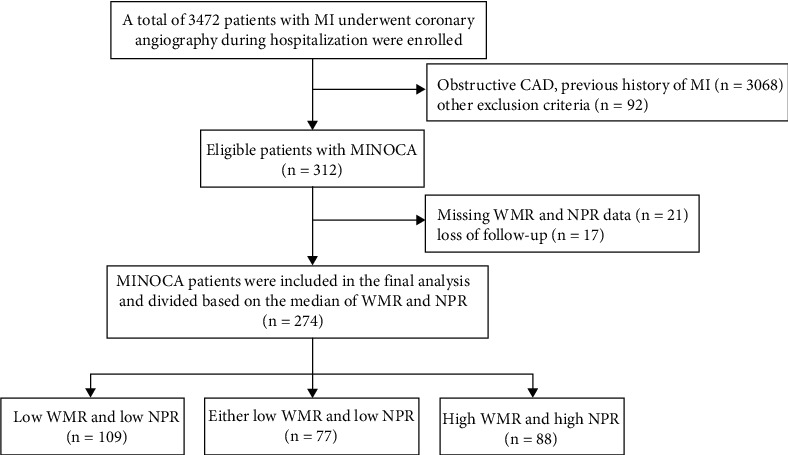
Flowchart of the study selection process. MI: myocardial infarction; CAD: coronary artery disease; MINOCA: myocardial infarction with nonobstructive coronary arteries; WMR: white blood cell count to mean platelet volume ratio; NPR: neutrophil-to-platelet ratio.

**Figure 2:**
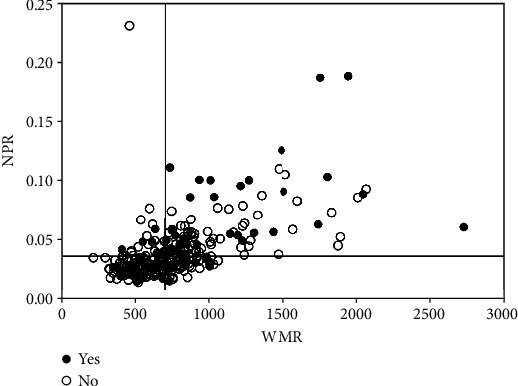
Scatterplot between WMR and NPR. WMR and NPR had a significant correlation (Spearman *r* = 0.613, *P* < 0.001). When WMR cut-off value of 701 and NPR cut-off value of 0.03 were used, 34% of MACE occurred in high WMR and high NPR group (right upper quadrant). WMR: white blood cell count to mean platelet volume ratio; NPR: neutrophil-to-platelet ratio; MACE: major adverse cardiovascular events.

A total of 3472 AMI patients were consecutively selected, of whom 312 patients (9.0%) satisfied the diagnostic criteria of MINOCA. Of these, 38 patients were excluded due to missing WMR and NPR data (*n* = 21) and loss of follow-up (*n* = 17). Finally, 274 MINOCA patients were enrolled in the final analysis of the present study (Figure 1). The mean age was 63.36 ± 13.41 years, of which 139 (50.7%) were female. The baseline demographic and biochemical data are summarized in Tables 1 and 2. The median values of WMR and NPR were 701 and 0.03, respectively. There was a significant correlation between WMR and NPR (Spearman *r* = 0.613, *P* < 0.001) (Figure 2). The participants were classified into three groups according to the median values of WMR and NPR, namely, high WMR and high NPR group (*n* = 88), either low WMR or low NPR group (*n* = 77), and low WMR and low NPR group (*n* = 109). Heart rate was higher in the high WMR and high NPR group than in other groups, while the level of LVEF was lower. Systolic blood pressure was higher in the low WMR and NPR groups than in the other groups. Angiographic data showed that the prevalence of mild coronary artery stenosis (<50% stenosis) was higher in either the low WMR or low NPR group when compared with other groups. There were no significant differences in age, gender, hypertension, diabetes, atrial fibrillation, hyperlipidemia, and smoking history among the three groups.

### 3.2. Clinical Outcomes Stratified by WMR and NPR

**Figure 3:**
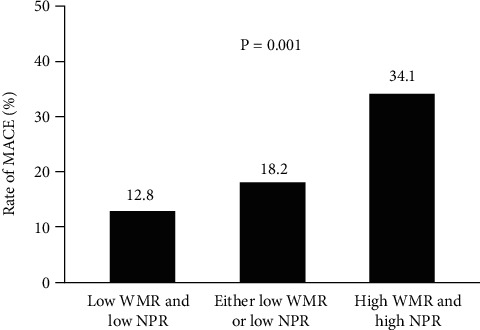
Rate of adverse events in MINOCA patients based on combined WMR and NPR medians. WMR: white blood cell count to mean platelet volume ratio; NPR: neutrophil-to-platelet ratio; MACE: major adverse cardiovascular events.

**Figure 4:**
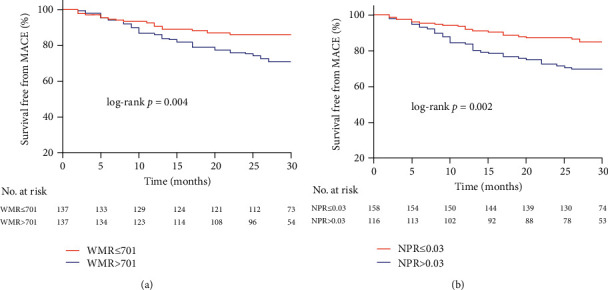
Kaplan-Meier survival curves for MACE in MINOCA patients with WMR (a) and NPR (b) median. WMR: white blood cell count to mean platelet volume ratio; NPR: neutrophil-to-platelet ratio; MACE: major adverse cardiovascular events.

**Figure 5:**
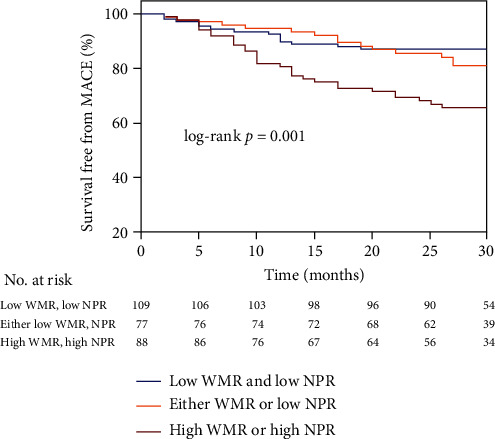
Kaplan-Meier survival curves for MACE in MINOCA patients with combined WMR and NPR median. WMR: white blood cell count to mean platelet volume ratio; NPR: neutrophil-to-platelet ratio; MACE: major adverse cardiovascular events.

**Figure 6:**
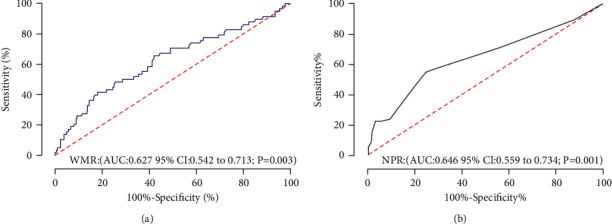
Receiver operating characteristic (ROC) curve of the ability of WMR (a) and NPR (b) to predict MACE in MINOCA patients. AUC: area under the curve; CI: confidence interval; WMR: white blood cell count to mean platelet volume ratio; NPR: neutrophil-to-platelet ratio.

During the median follow-up duration of 28 months, 58 incidences of MACE were recorded, in which in the low WMR and low NPR group, 14 MACE occurred (5 cardiovascular deaths, 0 nonfatal MI, 0 heart failure, 8 angina rehospitalizations, and 1 stroke), in the either low WMR or low NPR group, 14 MACE occurred (3 cardiovascular deaths, 0 nonfatal MI, 1 heart failure, 8 angina rehospitalizations, and 2 strokes), and in the high WMR and high NPR group, 30 MACE occurred (10 cardiovascular deaths, 1 nonfatal MI, 2 heart failures, 17 angina rehospitalizations, and 0 strokes). The MACE rate was significantly higher in patients with high WMR and NPR. The rates of MACE in high WMR and high NPR vs. either low WMR or low NPR vs. low WMR and low NPR groups were 34.1% vs. 18.2% vs. 12.8%, respectively, *P* = 0.001 (Figure 3). The Kaplan-Meier MACE-free survival curves demonstrated worse long-term outcomes in patients with high WMR (WMR >701) and patients with high NPR (NPR>0.03) (long-rank *P* = 0.004 and 0.002, respectively) (Figures 4(a) and 4(b)). Notably, the combined high WMR and NPR demonstrated a worse long-term prognosis than other groups (long-rank *P* = 0.001) (Figure 5). The ROC curve analysis for the prediction of clinical outcomes of NPR and WMR is shown in Figures 6(a) and 6(b). The area under the curve (AUC) of NPR and WMR for MACE showed that the WMR (AUC: 0.627, 95% CI: 0.542 to 0.713; *P* = 0.003) and NPR (AUC: 0.646, 95% CI: 0.559 to 0.734; *P* = 0.001) have moderate significant prediction of MACE.

### 3.3. Independent Predictors of MACE

To determine whether a correlation existed between WMR and NPR with MACE, we constructed three Cox hazard models (Table 3). Model A was performed with WMR as ≤701 vs. >701, model B with NPR as ≤0.03vs. >0.03, and model C with a combination of WMR and NPR as high WMR and high NPR, either low WMR or low NPR, and low WMR and low NPR. All models were adjusted for age, sex, smoking, hypertension, diabetes mellitus, hyperlipidemia, atrial fibrillation, LVEF, and the degree of coronary stenosis. In model A, WMR>701 was an independent risk predictor of MACE (adjusted HR, 2.155; 95% CI, 1.196 to 3.881; *P* = 0.011). In model B, NPR>0.03 was an independent predictor of MACE outcome (adjusted HR, 1.831; 95% CI, 1.036 to 3.235; *P* = 0.037). In model C, the combined high WMR and high NPR were independent predictors of MACE events with the highest hazard ratio (adjusted HR, 2.511; 95% CI, 1.271 to 4.960; *P* = 0.008).

Further analysis of the association between high NPR, WMR, and the risk of MACE stratified by sex, age, and comorbidities (e.g., hypertension, diabetes, smoking, and atrial fibrillation) was performed, which demonstrated that there were no interactions between WMR>701, NPR>0.03, and clinically related variables (all *P* for interaction >0.05) (Tables 4 and 5). That is, high NPR and high WMR were associated with an increased risk of MACE in all MINOCA patients, and this association was consistent throughout MINOCA subgroups.

## 4. Discussion

The current study was the first to demonstrate the association between WMR and NPR in predicting long-term outcomes in MINOCA patients. The main findings in the present study were as follows: (1) high WMR and high NPR were associated with worse long-term outcomes with the highest incidence of MACE; (2) WMR and NPR, both separately and in combination, are independent predictors of long-term outcomes among MINOCA populations.

Despite MINOCA's high prevalence and poor clinical outcomes, the management of these patients remained controversial mainly due to unclear etiology, risk factors, and limited evidence-based clinical studies. The critical management of MINOCA is based on identifying the etiologic risk factors and final diagnosis [2, 6, 29]. The pathophysiologic mechanism of different etiologies of MINOCA has been described, including atherosclerotic plaque disruption, coronary vasospasm, microvascular dysfunction, spontaneous coronary dissection, and supply-demand imbalance [2, 6]. The potential role of inflammation in the etiology of CAD, atherosclerotic coronary plaque instability, and thrombus formation has been established earlier [30, 31]. The components of complete blood count and the derived parameters, particularly WBC count, MPV, WMR, NPR, NLR, and PLR, have been evaluated as inflammation risk factors to explore their role and predictive ability in CAD. Therefore, inflammation may include a critical element in the initiation of patients with MINOCA.

A recent clinical study showed that inflammatory markers such as NLR, NPR, PLR, and C-reactive protein at admission were significantly higher in the MINOCA population [32]. Another large cohort study also demonstrated that acute MINOCA is accompanied by a specific inflammatory pattern, which is reflected by C-reactive protein levels [33]. However, data are scarce on the relationship between inflammatory biomarkers and clinical outcomes in this clinical entity. In a recent study, Gürdal et al. investigated the prognostic value of NLR in 72 patients with MINOCA and found that NLR was an independent predictor of mortality over a median follow-up of 21 months [34]. In this study, we explored the prognostic importance of the inflammatory biomarkers (WMR and NPR) and elucidated whether these biomarkers can provide any potential clinical significance in patients diagnosed with MINOCA.

Total WBC counts and platelet counts have previously been implicated in the development of cardiovascular disease [10, 11]. In patients with AMI [35], acute ischemic stroke [36], and heart failure with preserved ejection fraction [37], a high WBC count has been associated with significantly increased long-term mortality and poor clinical outcomes. Even in terms of short-term prognosis, an elevated WBC count is an independent predictor of death in patients with acute coronary syndromes [38]. In addition, Chung et al. also report that a high WBC count was associated with large infarct size and worse clinical outcomes in ST-segment elevation MI (STEMI) patients [35]. Larger size platelets are more dynamic than smaller sizes metabolically and enzymatically, and they show a higher thrombotic ability [39]. MPV accurately measures platelet volume [39], and it is a potentially valuable predictive biomarker of platelet reactivity in cardiovascular disorders [39, 40]. Systematic review and meta-analysis showed that elevated MPV is correlated with greater mortality in patients with AMI [40]. Likewise, patients with a high MPV are more likely to develop coronary artery disease than those with a low MPV [41]. High WBC and MPV levels also have been associated with impaired reperfusion in obstructed coronary arteries [42], as well as predicting impaired microvascular perfusion in patients with STEMI [43]. WMR, as a combination of WBC and MPV, recently emerged as a novel biomarker in predicting long-term adverse clinical outcomes both in patients with STEMI [18] and non-ST-elevation MI (NSTEMI) [19, 44] better than WBC and MPV separately. Higher WMR was associated with a significantly increased SYNTAX score in patient's diagnosis with NSTEMI [45]. In patients undergoing primary percutaneous coronary intervention, a greater WMR on admission was also correlated with an increased risk of clinical outcomes [46]. However, the clinical importance of WMR in MINOCA patients has not yet been explored. Our study demonstrated an association of WMR with long-term outcomes in MINOCA patients, which indicated that patients with high WMR have worse clinical outcomes than low WMR patients. Additionally, WMR was an independent risk factor of long-term adverse events among MINOCA populations even after adjusting for multiple clinical risk factors.

On the other hand, platelets have been indicated to participate in atherosclerotic plaque development, extension, and atherothrombosis, which play a critical role in ACS events through interaction with leukocytes [47, 48], and higher platelets were reported to be a predictor of cardiovascular mortality [49]. Neutrophils also have been reported to participate in microvascular injury and no-reflow state in AMI as mediators of acute inflammatory reaction [50]. NPR was recently introduced as a novel predictor of mortality in STEMI patients who underwent primary coronary revascularization to improve the strength of acute inflammatory response revealed by neutrophils and consider antecedent chronic inflammatory condition indicated by platelets [22], in which higher NPR was associated with two-fold increased risk 30-day mortality. To date, no previous studies have explored the prognostic importance of NPR in the MINOCA population. In our study, we found that the NPR was a significant predictor of MACE, and the patients with high NPR demonstrated worse clinical outcomes. Our findings extend prior studies by demonstrating that NPR was an independent risk factor for long-term adverse events even after adjusting for multiple clinical risk factors. Notably, when we investigated the relationship between combined WMR and NPR, we found that this combination was even stronger at predicting clinical outcomes in MINOCA patients. The rate of MACE was higher in patients with combined high WMR and high NPR and was more closely associated with MACE than in patients with both/or either low WMR and/or low NPR. Moreover, patients with both high WMR and high NPR had a 2.51-fold higher risk of adverse clinical outcomes. The association between these inflammatory biomarkers and worse clinical outcomes among patients with MINOCA may perhaps be due to the underlying pathophysiologic etiologies of this heterogeneous clinical entity which include plaque disruption and coronary thrombus [6].

Taken together, the findings of the present investigation are of significant clinical interest, which suggests that both WMR and NPR calculated from WBC subtype counts are readily and widely available inflammatory markers. The elevated levels of WBC, MPV, neutrophils, and platelets in acute MINOCA are indicative of a specific inflammatory pattern. The application of WMR and NPR may enhance clinical reasoning about the necessity of performing comprehensive risk stratification to reduce the MACE rate in MINOCA patients with high WMR and NPR. Large-scale prospective investigations are required to confirm our results and identify the impact of inflammatory biomarkers in the medical decisions in MINOCA populations.

### 4.1. Limitations

This study's findings need to be interpreted with a few limitations. First, it was an observationally retrospective, single-center, small sample size study; therefore, the data's generalizability is limited. Second, other inflammatory cytokines and oxidative stress markers were not available. Third, we used spot laboratory complete blood cell counts with the highest WBC count rather than blood test panel at a time interval. In addition, although we adjusted for potential confounding factors, we cannot eliminate unmeasured residual confounders. A prospective, multicenter study with time-interval blood cell counts, assessment of plaque burden, and evaluation of other inflammatory biomarkers is needed in future studies.

## 5. Conclusion

High WMR and high NPR separately or in combination were correlated with increased risk of MACE among MINOCA patients, suggesting WMR and NPR may assist as a reliable inflammatory marker in risk prediction of MINOCA patients.

## Figures and Tables

**Table 1:** Baseline characteristics based on the WMR and NPR.

	Low WMR and low NPR (*n* = 109)	Either low WMR or low NPR (*n* = 77)	High WMR and high NPR (*n* = 88)	*P* value
General characteristics				
Age (years)	64.06 ± 11.97	60.76 ± 13.14	64.76 ± 15.04	0.126
Female, *n* (%)	63 (57.8)	39 (50.6)	37 (42.0)	0.089
Vital signs				
SBP (mmHg)	144.72 ± 20.12	142.66 ± 23.54	136.22 ± 24.07	0.027
DBP (mmHg)	81.02 ± 10.38	81.97 ± 14.72	79.02 ± 13.39	0.307
HR (beats per minute)	82.47 ± 16.93	77.83 ± 14.07	84.68 ± 20.73	0.041
Risk factors, *n* (%)				
Hypertension	54 (49.5)	43 (55.8)	40 (45.5)	0.409
Diabetes	18 (16.5)	9 (11.7)	20 (22.7)	0.167
Smoking history	41 (37.6)	26 (33.8)	42 (47.7)	0.158
Atrial fibrillation	13 (11.9)	6 (7.8)	10 (11.4)	0.638
Hyperlipidemia	10 (9.2)	16 (20.8)	14 (15.9)	0.080
LVEF (%)	56.79 ± 10.57	56.07 ± 9.78	51.01 ± 13.55	0.002
Angiographic data, *n* (%)				
Mild coronary stenosis	47 (43.1)	52 (67.5)	55 (62.5)	0.001

Abbreviations: WMR: white blood cell count to mean platelet volume ratio; NPR: neutrophil-to-platelet ratio; SBP: systolic blood pressure; DBP: diastolic blood pressure; HR: heart rate; LVEF: left ventricular ejection fraction.

**Table 2:** Biochemical data based on the WMR and NPR.

	Low WMR and low NPR (*n* = 109)	Either low WMR or low NPR (*n* = 77)	High WMR and high NPR (*n* = 88)	*P* value
WBC, ×10^9^/L	5.83 ± 1.04	7.97 ± 1.35	12.19 ± 4.0	< 0.001
Neutrophil, ×10^9^/L	3.71 ± 0.95	5.54 ± 1.19	9.99 ± 3.92	< 0.001
Lymphocyte, ×10^9^/L	1.62 ± 0.55	1.78 ± 0.76	1.50 ± 0.85	0.050
Platelets, ×10^9^/L	197.10 ± 51.26	214.04 ± 73.68	188.0 ± 55.40	0.019
MPV (fl)	11.20 ± 1.23	10.80 ± 1.25	11.01 ± 1.39	0.115
WMR	526.32 ± 103.82	748.59 ± 152.61	1127.55 ± 406.15	< 0.001
NPR	0.02 ± 0.01	0.03 ± 0.02	0.06 ± 0.03	< 0.001
NLR	2.74 ± 1.69	4.13 ± 3.66	8.97 ± 6.31	< 0.001
PLR	138.68 ± 64.29	140.41 ± 82.14	157.18 ± 77.05	0.176

Abbreviations: WBC: white blood cell counts; MPV: mean platelet volume; WMR: WBC to MPV ratio; NPR: neutrophil-to-platelet ratio; NLR: neutrophil-to-lymphocyte ratio; PLR: platelet-to-lymphocyte ratio.

**Table 3:** Results of Cox proportional-hazards regression analysis.

	Hazard ratio	95% CI	*P* value
Model A∗—WMR ≤ 701 vs. > 701			
WMR>701	2.155	1.196-3.881	0.011
Age	1.031	1.007-1.056	0.012
AF	2.286	1.108-4.715	0.025
LVEF	0.979	0.959-0.999	0.052
Model B∗—NPR ≤ 0.03 vs. > 0.03			
NPR>0.03	1.831	1.036-3.235	0.037
Age	1.028	1.003-1.054	0.030
LVEF	0.978	0.958-0.999	0.038
Model C∗—combination of WMR and NPR			
Age	1.027	1.002-1.053	0.033
Low WMR and low NPR	Reference	Reference	
Either low WMR or low NPR	1.479	0.673-3.248	0.330
High WMR and high NPR	2.511	1.271-4.960	0.008

Abbreviations: WMR: white blood cell count to mean platelet volume ratio; NPR: neutrophil-to-platelet ratio; AF: atrial fibrillation; LVEF: left ventricular ejection fraction; CI: confidence interval. ∗Models adjusted for age, sex, smoking, hypertension, diabetes mellitus, hyperlipidemia, atrial fibrillation, left ventricular ejection fraction, and the degree of coronary stenosis.

**Table 4:** Subgroup analysis of the association between high WMR and MACE.

Factors	Subgroup	HR (95% CI)	Interaction *P* value
Age	**<**65 years	3.048 (1.227-7.571)	0.263
	≥65 years	1.026 (0.381-2.764)	
Sex	Male	3.207 (1.300-7.913)	0.257
	Female	1.663 (0.807-3.425)	
Hypertension	Yes	2.605 (1.245-5.451)	0.462
	No	1.709 (0.748-3.908)	
Diabetes	Yes	1.743 (0.584-5.202)	0.664
	No	2.292 (1.215-4.325)	
Smoking	Yes	3.426 (1.278-9.179)	0.232
	No	1.673 (0.845-3.314)	
Atrial fibrillation	Yes	2.795 (0.846-9.226)	0.710
	No	2.305 (1.233-4.307)	

Abbreviations: HR: hazard ratio; CI: confidence interval.

**Table 5:** Subgroup analysis of the association between high NPR and MACE.

Factors	Subgroup	HR (95% CI)	Interaction *P* value
Age	**<**65 years	2.037 (0.880-4.716)	0.890
	≥65 years	2.200 (1.113-4.345)	
Sex	Male	2.430 (1.099-5.372)	0.832
	Female	2.167 (1.057-4.443)	
Hypertension	Yes	2.136 (1.085-4.206)	0.710
	No	2.621 (1.121-6.126)	
Diabetes	Yes	3.777 (1.051-13.562)	0.353
	No	1.909 (1.054-3.457)	
Smoking	Yes	2.545 (1.089-5.951)	0.677
	No	2.044 (1.038-4.023)	
Atrial fibrillation	Yes	1.641 (0.479-5.616)	0.638
	No	2.289 (1.278-4.101)	

Abbreviations: HR: hazard ratio; CI: confidence interval.

## Data Availability

The raw data supporting the conclusion of this manuscript will be made available by the corresponding author on request.
